# Impact of Short-Term Liraglutide Therapy on Non-Invasive Markers of Liver Fibrosis in Patients with MASLD

**DOI:** 10.3390/metabo15080510

**Published:** 2025-07-31

**Authors:** Aleksandra Bołdys, Maciej Borówka, Łukasz Bułdak, Bogusław Okopień

**Affiliations:** Department of Internal Medicine and Clinical Pharmacology, Faculty of Medical Sciences in Katowice, Medical University of Silesia, Medyków 18, 40-752 Katowice, Poland; d201308@365.sum.edu.pl (M.B.); lbuldak@sum.edu.pl (Ł.B.); bokopien@sum.edu.pl (B.O.)

**Keywords:** MASLD, GLP-1 analog, obesity, non-invasive fibrosis markers

## Abstract

**Background/Objectives**: Affecting close to one-third of the global population, metabolic dysfunction-associated steatotic liver disease (MASLD) is a highly prevalent chronic liver disorder linked to metabolic risk factors such as obesity and insulin resistance. Liver fibrosis is a key determinant of prognosis, and its progression increases the risk of liver-related and overall mortality. This exploratory research evaluated the potential impact of a 3-month intervention involving dietary counseling and liraglutide therapy on liver fibrosis and related metabolic markers in patients with MASLD and obesity without diabetes. **Methods**: In this prospective, single-arm exploratory intervention, 28 adult patients with MASLD and obesity received structured dietary counseling and daily subcutaneous liraglutide for 12 weeks. Liver fibrosis was assessed using non-invasive indices (FIB-4, APRI, BARD, ELF) and transient elastography performed with the FibroScan^®^ device (Echosens, Paris, France). **Results**: After 3 months, a significant reduction in liver stiffness (−7.14%, *p* < 0.05) and ELF score (from 6.71 to 6.63; −1.2%, *p* < 0.05) was observed. APRI (*p* = 0.06) and FIB-4 (*p* = 0.09) showed trends toward improvement, while the BARD score and AST/ALT ratio remained unchanged. **Conclusions:** Short-term liraglutide therapy combined with lifestyle modification may improve early-stage liver fibrosis in patients with MASLD and obesity, as indicated by reductions in liver stiffness and ELF score. These preliminary findings highlight the potential of advanced non-invasive fibrosis markers in monitoring treatment response. However, as an exploratory study, results should be interpreted with caution, and larger, long-term trials are needed to confirm these observations and evaluate efficacy in patients with more advanced fibrosis stages.

## 1. Introduction

With a global prevalence nearing one-third of the population and a growing incidence, MASLD (metabolic dysfunction-associated steatotic liver disease) has emerged as a critical concern in hepatology and metabolic medicine [1]. MASLD is closely linked with other metabolic disorders such as overweight, obesity, and type 2 diabetes mellitus (T2DM), which are both risk factors and clinical manifestations of the disease. This diagnosis holds particular clinical importance because of its association with elevated all-cause mortality, estimated at 12.6 (95% CI: 6.68–23.67) per 1000 person-years, and liver-specific mortality estimated at 0.92 (95% CI: 0.00–2.21) per 1000 person-years [2,3,4]. MASLD is defined by the presence of hepatic steatosis—confirmed by imaging or histology—in conjunction with at least one cardiometabolic risk factor, including overweight or obesity, increased waist circumference, type 2 diabetes, or atherogenic dyslipidemia. The term MASLD encompasses a spectrum of liver pathology ranging from simple steatosis (MASL) to steatohepatitis (MASH), which is histologically characterized by hepatocellular ballooning and lobular inflammation, and may progress to advanced fibrosis or cirrhosis. This terminology replaces the former classification of non-alcoholic fatty liver disease (NAFLD) and is embedded within the broader consensus definition of steatotic liver disease (SLD), which also includes MASLD with moderate alcohol consumption (MetALD), alcohol-related liver disease (ALD), and other specific or cryptogenic etiologies [5]. Ultrasound and transient elastography (FibroScan^®^) are recommended by the 2024 EASL guidelines as part of non-invasive diagnostic pathways for MASLD, with ultrasound serving as an accessible screening tool for hepatic steatosis and FibroScan^®^ providing more accurate quantification of both steatosis (via controlled attenuation parameter) and fibrosis (via liver stiffness measurement), although neither method enables definitive diagnosis of MASH, which still requires histological assessment [5].

One of the key clinical manifestations of MASLD is liver fibrosis, typically staged from F0 (no fibrosis) to F4 (cirrhosis). Fibrosis progression, particularly to stage F3, significantly increases the risk of adverse hepatic outcomes—up to fivefold higher compared with F0 [6,7].

Given the growing burden of MASLD, several international societies have issued guidelines on its diagnosis and treatment, including the European Association for the Study of the Liver (EASL), European Association for the Study of Diabetes (EASD), European Association for the Study of Obesity (EASO), and the American Association for the Study of Liver Diseases (AASLD) [5]. Up to date, resmetirom is a uniquely approved medication by the U.S. Food and Drug Administration for the therapy of steatohepatitis, whereas no pharmacological therapy is yet fully approved by the European Medicines Agency (EMA) for this indication. However, on 19 June 2025, the Committee for Medicinal Products for Human Use (CHMP) adopted a positive opinion recommending the granting of a conditional marketing authorization for resmetirom for the treatment of adults with noncirrhotic metabolic dysfunction-associated steatohepatitis (MASH) [8,9].

Among the most promising candidates for MASLD-related treatment are glucagon-like peptide-1 receptor agonists (GLP-1 RAs), given their proven efficacy in the management of obesity and T2DM–key pathogenic drivers of MASLD. In addition to their metabolic effects, GLP-1 RAs exert pleiotropic benefits on the cardiovascular system and kidneys. The hepatoprotective effects of GLP-1 receptor agonists may be explained by a reduction in hepatic steatosis via decreased de novo lipogenesis, improved hepatic insulin sensitivity, and attenuation of inflammation and oxidative stress [10,11,12].

Although several small-scale studies have suggested potential benefits of GLP-1 RAs on liver histology, current guidelines (EASL–EASD–EASO and AASLD) do not recommend GLP-1 agonists specifically for the treatment of MASH. However, they are considered safe for patients with MASLD and are recommended when obesity or T2DM coexists, which often justifies their clinical use [5,13].

Although resmetirom is currently undergoing regulatory review in Europe (with the final decision by the EMA expected in August 2025 [14]), further studies are needed to explore additional or complementary therapeutic strategies, particularly for patients who may not qualify for or respond to this treatment. Notably, our study was designed and initiated prior to the approval of resmetirom in the United States and before the regulatory process began in Europe. Accordingly, we carried out a prospective, exploratory interventional study to evaluate the effectiveness of a 3-month weight management program consisting of standard dietary counseling combined with the start of GLP-1 receptor agonist therapy (liraglutide). We aimed to assess its impact on liver fibrosis stage, subclinical disease markers, and prognostic algorithms used in the clinical management of patients with MASLD. We hypothesized that this combined approach would result in measurable improvements in non-invasive markers of liver fibrosis and metabolic dysfunction.

## 2. Materials and Methods

### 2.1. Subjects

The study population consisted of 28 obese adults (BMI ≥ 30 kg/m^2^), including 19 females and 9 males, with no reported comorbidities, all of whom fulfilled the inclusion criteria for treatment with subcutaneous liraglutide administered once daily in a dose-escalation protocol (from 0.6 mg to 3.0 mg per day). The mean age of participants was 49.1 ± 11.6 years, with an age range of 26 to 78 years. In addition to pharmacological therapy, all participants received standardized dietary counseling based on national guidelines for obesity management, accompanied by printed materials summarizing recommended physical activity and exercise examples appropriate for patients with hepatic steatosis [15,16]. Subjects were recruited from the Outpatient Clinic of the Department of Internal Medicine and Clinical Pharmacology, Medical University of Silesia, Katowice, Poland. Informed written consent was obtained from each patient prior to inclusion. Venous blood samples were collected at two time points: before initiation of liraglutide (baseline) and following three months of treatment. All enrolled individuals completed the intervention period. The study protocol received approval from the Ethics Committee of the Medical University of Silesia (BNW/NWN/0052/KB1/97/23).

### 2.2. Inclusion and Exclusion Criteria

Participants were eligible for inclusion in the study if they met the following criteria: (a) age of 18 years or older; (b) provision of written informed consent; (c) diagnosis of obesity, defined as a body mass index (BMI) ≥ 30 kg/m^2^; and (d) presence of hepatic steatosis confirmed by ultrasound imaging at the initial assessment.

Exclusion criteria included the following: (a) age below 18 years; (b) inability or unwillingness to cooperate with study procedures; (c) absence of informed consent; (d) pregnancy or breastfeeding; (e) known hypersensitivity to GLP-1 receptor agonists such as semaglutide or dulaglutide; (f) history of diabetic ketoacidosis; (g) current or past alcohol or psychoactive substances (including nicotine) dependence; (h) presence of acute or chronic inflammatory disorders; (i) clinically significant abnormalities in laboratory tests—defined as estimated glomerular filtration rate (eGFR) < 60 mL/min/1.73 m^2^, ALT or AST > 3 times the upper limit of normal (ULN), total bilirubin > 1.2 mg/dL, hemoglobin < 10 g/dL or >16 g/dL, red blood cell count (RBC) < 3.5 million/μL or >5.5 million/μL, white blood cell count (WBC) < 3.5 thousand/μL or >10 thousand/μL, and platelet count (PLT) < 140 thousand/μL or >400 thousand/μL; (j) ongoing treatment with any GLP-1 receptor agonist; and (k) diagnosis of malignancy within the five years preceding study enrollment.

### 2.3. Anthropometric and Laboratory Assessments

Eligible participants underwent a comprehensive physical examination and anthropometric assessment, including measurements of height, body weight, and waist-to-hip ratio (WHR). Following this, venous blood samples were collected for laboratory evaluation. Specifically, 2 mL of whole blood was used for complete blood count analysis, and 5 mL of blood was collected for biochemical profiling. The biochemical panel included assessments of electrolytes (sodium, potassium), renal function markers (uric acid, urea, creatinine with estimated glomerular filtration rate [eGFR] calculation), liver function tests (total bilirubin, aspartate aminotransferase [AST], alanine aminotransferase [ALT], gamma-glutamyltransferase [GGT], alkaline phosphatase [ALP]), glucose, lipid profile (total cholesterol, low-density lipoprotein [LDL], high-density lipoprotein [HDL], triglycerides [TG]), thyroid-stimulating hormone (TSH), ferritin, serum iron, and total iron-binding capacity (TIBC).

Laboratory analyses were performed using the Sysmex XN-1000 hematology analyzer (Sysmex Corporation, Kobe, Japan) for complete blood count, and the Cobas PRO 9 (Roche Diagnostics, Basel, Switzerland) analytical system for biochemical assays.

Additionally, serum (5 mL) and plasma (4 mL) samples were separated, aliquoted, and stored at −70 °C for future analysis of established and exploratory biomarkers associated with liver fibrosis. These included hyaluronic acid (HYA), N-terminal propeptide of type III procollagen (PIIINP), tissue inhibitor of metalloproteinases-1 (TIMP-1), hepatocyte growth factor (HGF), chitinase-3-like protein 1 (CHI3L1), and transforming growth factor-alpha (TGF-α). All ELISA-based measurements were performed using serum samples stored in a single freeze–thaw cycle.

Quantification of serum biomarkers was conducted using commercially available enzyme-linked immunosorbent assay (ELISA) kits (Cloud-Clone Corp., Houston, TX, USA), following the manufacturer’s protocols. All assays were conducted in duplicate, and absorbance was measured at 450 nm using the xMark™ Microplate Absorbance Reader (Bio-Rad Laboratories, Hercules, CA, USA). Kits were validated according to internal standards, with intra-assay coefficients of variation (CV) below 10% and inter-assay CVs not exceeding 12%, and detection ranges within expected physiological concentrations (as per manufacturer specifications). For the quantification of PIIINP, TIMP-1, and HGF, 100 μL of serum sample or standard was added to each well and incubated for 1 h at 37 °C. After aspiration, 100 μL of detection reagent A was added and incubated for another hour at 37 °C, followed by three washes with wash buffer. Next, 100 μL of detection reagent B was added and incubated for 30 min at 37 °C. After five additional washes, 90 μL of substrate solution was added and incubated for 10–20 min at 37 °C, protected from light. The reaction was terminated by the addition of 50 μL of stop solution, and absorbance was measured immediately. For HYA quantification, 50 μL of sample or standard and 50 μL of detection reagent A were sequentially added to each well, mixed, and incubated for 1 h at 37 °C. The wells were then washed three times before adding 100 μL of detection reagent B, followed by a 30 min incubation at 37 °C. After five additional washes, 90 μL of substrate solution was added and incubated for 10–20 min at 37 °C, followed by the addition of 50 μL of stop solution and immediate absorbance measurement. For CHI3L1 and TGF-α, 100 μL of sample, blank, or standard was added to each well, covered with a plate sealer, and incubated for 1 h at 37 °C. Without washing, 100 μL of detection reagent A was added, followed by a second 1 h incubation. Wells were then washed three times using a 1× wash buffer, with a 1–2 min soak during each wash. After removing residual liquid, 100 μL of detection reagent B was added, and plates were incubated for 30 min at 37 °C. Five additional washes were performed before adding 90 μL of substrate solution and incubating for 10–20 min at 37 °C, protected from light. Finally, 50 μL of stop solution was added, and absorbance was recorded immediately at 450 nm.

### 2.4. Liver Fibrosis Assessment

Liver fibrosis was evaluated using transient elastography, a non-invasive imaging technique performed in our study with the FibroScan^®^ 430 Mini+ device (Echosens, Lyon, France). This elastography method quantifies liver stiffness, an indirect marker of fibrosis, expressed in kilopascals (kPa).

Following a minimum five-minute rest, examinations were carried out with the patient supine, the right arm placed behind the head, and the legs positioned with the right crossed over the left. The probe was applied at the right mid-axillary line, in the intercostal space corresponding to the level of the xiphoid process. Multiple initial measurements were performed to determine the most suitable acoustic window, followed by the acquisition of ten valid measurements. These values were subsequently used to calculate median liver stiffness, which served as the final parameter for fibrosis assessment.

Quantification of liver fibrosis was based on established reference cut-off values specific to metabolic dysfunction-associated steatotic liver disease [5,17]. Liver stiffness values between 2.0 and 7.0 kPa were interpreted as no-to-mild fibrosis (stage F0–F1), values from 7.5 to 10.0 kPa indicated moderate fibrosis (stage F2), values from 10.1 to 13.9 kPa were consistent with advanced fibrosis (stage F3), and values equal to or above 14.0 kPa were considered diagnostic of cirrhosis (stage F4). These thresholds are consistent with published data on steatotic liver disease of metabolic origin, although some variability exists depending on the etiology of liver disease, patient characteristics, and the specific device used. The lower limit for advanced fibrosis is generally accepted to begin just above 10 kPa, though some sources place it at 10.1 or 11.0 kPa [18,19,20]. In our study, we applied the commonly used range of 10.1 to 13.9 kPa for identifying stage F3 fibrosis, while acknowledging the potential overlap between stages in clinical settings.

According to the 2024 European Association for the Study of the Liver (EASL) Clinical Practice Guidelines on metabolic dysfunction-associated steatotic liver disease, a liver stiffness value below 8.0 kPa can be used to rule out advanced fibrosis in most patients. In our study, none of the participants had liver stiffness values consistent with advanced fibrosis or cirrhosis. All individuals were classified as having fibrosis stage F0–F1, based on liver stiffness values below 7.0 kPa.

Additionally, our study employed both widely used non-invasive blood-based scoring systems and less commonly applied indices, some of which are limited in clinical use due to availability or cost. These included the AST to Platelet Ratio Index (APRI), which utilizes the ratio of AST to platelet count [21]; the BARD score, which integrates BMI, the AST/ALT ratio and the presence of diabetes mellitus [22]; and the Fibrosis-4 (FIB-4) index, which is derived from age, AST, ALT, and platelet count [23]. Additionally, the Enhanced Liver Fibrosis (ELF) test combining three serum biomarkers associated with liver (HYA, PIIINP, and TIMP-1) [24] was utilized as well.

### 2.5. Statistical Analysis

The sample size was calculated based on an expected 10% reduction in liver steatosis, assuming a baseline CAP value of 300 ± 25 dB/m, with a significance level of 0.05 and power of 80%, yielding a minimum of 22 patients. No formal a priori sample size calculation was performed specifically for liver fibrosis, as the available literature at the time did not provide sufficiently consistent or quantifiable data to support a reliable estimation of the expected treatment effect or variability in this parameter. Therefore, fibrosis-related endpoints were treated as exploratory and interpreted accordingly. Statistical analyses were performed using Statistica software, version 13.3 (TIBCO Software Inc., 2017; Palo Alto, CA, USA). The normality of data distribution was assessed using the Shapiro–Wilk test. Variables with normal distribution are presented as mean ± standard deviation (SD), while non-normally distributed data are reported as median with interquartile range (IQR). Pre- and post-treatment comparisons were conducted using either the paired Student’s *t*-test for normally distributed variables or the Wilcoxon signed-rank test for non-parametric data. A *p*-value < 0.05 was considered indicative of statistical significance.

## 3. Results

### 3.1. Baseline Characteristic and Impact of Liraglutide Therapy on Anthropometric and Basic Laboratory Parameters

A total of 28 participants (19 females and 9 males) were enrolled in the study. Baseline and post-intervention characteristics are presented in Table 1 and Table 2.

Participants were eligible if they were ≥18 years old, had a BMI ≥ 30 kg/m^2^, hepatic steatosis confirmed by ultrasound, and provided informed consent. Exclusion criteria included pregnancy or breastfeeding, history of GLP-1 hypersensitivity or diabetic ketoacidosis, substance dependence (including nicotine), significant laboratory abnormalities (e.g., eGFR < 60 mL/min/1.73 m^2^, ALT/AST > 3 × ULN), active malignancy within 5 years, inflammatory disorders, or prior/current use of GLP-1 receptor agonists.

### 3.2. The Impact of Liraglutide Treatment on Liver Fibrosis (FibroScan^®^)

Patients with obesity exhibited mild hepatic fibrosis as determined by transient elastography (FibroScan^®^), with liver stiffness measurements (E) showing a median value of 5.60 kPa, corresponding predominantly to stage 1 fibrosis (F1). Following treatment with liraglutide, a meaningful decrease in liver stiffness was noted (−7.14%, *p* < 0.05), with the median E value decreasing to 5.20 kPa. However, despite this reduction, the fibrosis stage classification remained unchanged (Table 3).

### 3.3. The Effect of Liraglutide Therapy on Liver Fibrosis as Assessed by Diagnostic Algorithms

FibroScan^®^ is a highly effective non-invasive modality for evaluating both hepatic lipid accumulation and liver fibrosis. However, its widespread clinical application may be constrained by its relatively high cost, which highlights the need for more accessible non-invasive tools. 

The impact of liraglutide therapy on non-invasive diagnostic tools is detailed in Table 4.

Regarding liver fibrosis, only one algorithm, the ELF score, showed a statistically significant improvement over the 3-month treatment period, decreasing from 6.71 to 6.63 (−1.2%, *p* < 0.05). The APRI demonstrated a trend toward improvement (*p* = 0.06), although the change was not statistically significant.

### 3.4. Established and Exploratory Markers in Liver Fibrosis 

The following known and exploratory laboratory markers have been investigated in this study and the effects of liraglutide therapy on them are presented in Table 5.

Interestingly, although the ELF score significantly improved during the intervention, its three component serum biomarkers (HYA, TIMP-1, and PIIINP) remained unchanged. However, PIIINP changed its concentrations during the intervention from a median value of 243.80 ng/mL to 192.75 ng/mL, showing a trend toward marginal significance (*p* = 0.06). Furthermore, none of HGF, CHI3L1, or TGF-α showed significant improvement.

### 3.5. Adverse Effects and Treatment’s Safety

Throughout the 3-month intervention period, no adverse events such as acute pancreatitis or acute abdominal pain were documented. Mild, transient nausea occurred in five patients during the initial phase of therapy and resolved spontaneously during titration to the target dose of liraglutide.

## 4. Discussion

Metabolic dysfunction-associated steatotic liver disease (MASLD) is a widespread and clinically significant disorder closely accompanying obesity and type 2 diabetes mellitus (T2DM). Liver fibrosis is one of its key prognostic factors, yet no anti-fibrotic pharmacotherapy is currently approved in Europe. Although regulatory progress has recently been made with resmetirom, therapeutic options remain limited. Given the growing burden of MASLD and limited treatment options, glucagon-like peptide-1 receptor agonists (GLP-1 RAs), such as liraglutide, have emerged as promising therapeutic candidates due to their metabolic and potential hepatoprotective effects. In this context, we evaluated the impact of a combined intervention—liraglutide therapy and dietary counseling—on fibrosis and metabolic markers in patients with MASLD.

In our study, the effect of liraglutide on liver fibrosis was assessed using non-invasive predictive models based on laboratory parameters, as well as liver stiffness measurements obtained via elastography. These methods are currently recommended for routine clinical decision-making due to their accessibility and non-invasive nature [25,26]. During the study, liraglutide was the only GLP-1 receptor agonist available and approved in local market for obesity treatment.

As expected, the intervention resulted in a significant reduction in body weight and an improvement in carbohydrate metabolism, reflected by decreased levels of glycated hemoglobin and fasting glucose. This suggests good adherence to therapeutic recommendations among participants, which aligns with results from the same cohort, previously analyzed with regard to hepatic steatosis [27]. Regarding the fibrosis-4 (FIB-4) index, a widely recommended tool for initial fibrosis risk stratification [23], our study did not demonstrate a statistically significant change, although a non-significant trend toward reduction was observed (*p* = 0.09). None of the individual FIB-4 components changed significantly following treatment; however, a non-significant trend toward a decrease in ALT activity was noted (*p* = 0.07). It is worth noting that the baseline FIB-4 index in our population was relatively low (median 0.84), with most values below the 1.3 threshold indicating the need for further fibrosis evaluation. After treatment, this threshold was crossed downward in 4 out of 28 participants, and upward in only one. These findings are consistent with a randomized controlled trial (RCT) that also did not demonstrate a significant reduction in FIB-4 with liraglutide [28], supporting the notion that longer interventions or larger cohorts may be needed to detect meaningful effects. Nonetheless, some prospective [29] and retrospective studies [30] have reported such improvements. It is therefore possible that the limited sample size and short duration of our study contributed to the lack of statistical significance.

Regarding simple non-invasive indices of liver fibrosis, the APRI score exhibited a non-significant trend toward improvement over the 3-month treatment period (*p* = 0.06), which should be interpreted with caution due to the small sample size. This is consistent with the findings of a previously mentioned RCT, which did not demonstrate a significant effect of liraglutide on APRI [28]. However, two retrospective studies reported reductions in this index following liraglutide treatment [30,31]. Further investigation is warranted to determine the potential impact of GLP-1 receptor agonists on APRI and related fibrosis markers.

No significant differences were observed in the BARD index before and after liraglutide therapy. This index, which is rarely assessed in GLP-1 studies, uses a dichotomous scoring system, which may limit its sensitivity to detect change in short-duration interventions and small study samples. Similarly, our study did not demonstrate a significant change in the AST/ALT ratio. This aligns with previous findings from a prospective liraglutide study [32], although a combined study on semaglutide and dulaglutide did report a reduction in this ratio [33].

We also evaluated the effect of liraglutide on less commonly used laboratory fibrosis markers, including the ELF score. A modest but statistically significant reduction in the ELF index was observed, although none of its individual components changed significantly; only a non-significant trend toward a decrease in PIIINP concentration was noted (*p* = 0.06). These findings remain exploratory and warrant confirmation in larger trials. Since this model is based on biomarkers that are not routinely assessed in clinical practice, evidence regarding the effect of liraglutide on the ELF score remains limited, particularly in non-diabetic populations. One RCT [34] semaglutide reported a non-significant reduction in ELF score (*p* = 0.12) and a decrease in PIIINP levels, while a smaller study evaluating liraglutide showed a similar trend [35]. Our findings contribute to the growing body of exploratory evidence suggesting a possible influence of liraglutide on advanced fibrosis markers such as ELF and PIIINP. Notably, most existing studies in this field employed longer intervention periods, which may explain the more pronounced effects reported compared to our 3-month study. Importantly, the observed reduction in liver stiffness and ELF score may reflect early improvements in hepatic fibrogenesis and extracellular matrix remodeling, indicating a possible slowing of fibrotic progression in early-stage MASLD. Patients treated with GLP-1 receptor agonists in a real-world setting showed a significant decrease in liver stiffness associated with metabolic improvements, further supporting the clinical utility of elastography in monitoring MASLD progression [36,37]. Additionally, trials such as the LEAN study [38] demonstrated that reductions in ELF score correlate with histological improvement in fibrosis, reinforcing its role as a non-invasive marker of fibrosis regression.

With regard to liver stiffness measured via transient elastography, our study demonstrated a statistically significant reduction (−1.2%, *p* < 0.05). This is consistent with multiple studies evaluating both liraglutide and semaglutide [29,38,39,40,41,42]. However, no significant change was observed in fibrosis staging. It is important to note that our study population was relatively homogeneous, with the vast majority of participants presenting with stage F0 or F1 fibrosis and no cases of advanced fibrosis (F3 or F4). It is therefore plausible that studies involving patients with more severe baseline fibrosis may better reveal potential anti-fibrotic effects of liraglutide.

A key strength—but also a notable limitation—of this exploratory study was the relatively short intervention duration (3 months). Although we observed significant in selected non-invasive fibrosis markers within this time frame, it is likely that longer treatment periods (e.g., 6–12 months), as used in other trials, are required to fully capture the therapeutic potential of liraglutide in reversing or halting fibrosis progression.

Another important limitation was the absence of histological confirmation via liver biopsy, the current gold standard for fibrosis assessment. However, the invasiveness, cost, and ethical considerations associated with biopsy—particularly in non-cirrhotic, asymptomatic patients—rendered its use infeasible in our clinical setting. Moreover, liver biopsy is not routinely used in the diagnostic or therapeutic management of MASLD. While advanced imaging techniques such as magnetic resonance elastography could have strengthened our diagnostic approach, they were unavailable due to institutional resource constraints.

An additional limitation of this exploratory study was the absence of a placebo or comparative control group (e.g., lifestyle modification alone), which limits the attribution of observed effects specifically to liraglutide. This decision was driven by ethical considerations, as all participants expressed a willingness to initiate active treatment, having previously undergone unsuccessful or only partially effective lifestyle-based interventions. In this context, withholding pharmacological therapy in individuals with confirmed hepatic steatosis and obesity was deemed clinically and ethically inappropriate. While this approach aligned with real-world treatment preferences, it reduces the strength of causal inference regarding liraglutide’s independent effects.

Furthermore, the study was limited to liraglutide as the only GLP-1 receptor agonist, primarily due to restricted access to alternative agents during the study period and financial constraints among participants. These limitations also contributed to the relatively small sample size, potentially affecting statistical power and generalizability. Nevertheless, consistent trends across non-invasive fibrosis markers support the internal validity of the findings and justify further investigation.

Future studies are planned with larger cohorts and the inclusion of a comparative control group, supported by the expanded availability of GLP-1 receptor agonists in current clinical settings.

## 5. Conclusions

In this prospective study, a 3-month intervention with liraglutide combined with dietary counseling led to a significant improvement in selected non-invasive markers of liver fibrosis in patients with MASLD. Notably, a reduction in liver stiffness assessed by transient elastography and a decrease in the APRI and ELF scores were observed, despite the short duration of the intervention and relatively low baseline fibrosis stages in most participants. Although no statistically significant changes were found in other commonly used fibrosis indices such as FIB-4, BARD, or AST/ALT ratio, trends toward improvement were noted.

These findings suggest that liraglutide may exert beneficial effects on early-stage liver fibrosis, particularly when combined with structured weight management. Importantly, the results underscore the added value of advanced, non-invasive tools—such as FibroScan^®^ and the ELF score—in detecting clinically relevant fibrosis that may go unnoticed when relying solely on conventional indices. Although they have relatively high costs and limited accessibility in routine practice, these methods represent useful tools for identifying early fibrotic changes and monitoring treatment response. While scores like FIB-4 remain helpful for initial risk stratification, their limited sensitivity highlights the need for broader implementation of more precise biomarkers in both research and clinical practice.

As an exploratory study, these preliminary results should be interpreted cautiously. Further research with longer treatment durations, larger cohorts, and patients at more advanced stages of fibrosis are warranted to validate these findings and better define the anti-fibrotic potential of GLP-1 receptor agonists in MASLD.

## Figures and Tables

**Table 1:** Baseline demographic profile of the study population before and after the study.

	Number of Patients	Age—Years (Mean ± SD)	BMI Before—kg/m^2^ (Mean ± SD or Median; Q1, Q3)	BMI After—kg/m^2^ (Mean ± SD or Median; Q1, Q3)	*p*-Value	BW Before—kg (Mean ± SD)	BW After—kg (Mean ± SD)	*p*-Value
Total	28	49.1 ± 11.6	35.63 ± 5.10	33.81 ± 4.97	<0.001	102.32 ± 17.53	97.41 (89.28; 100)	<0.001
Women	19	49.9 ± 12.9	35.27 ± 4.72	33.5 ± 4.65	<0.001	96.68 ± 11.58	91.86 ± 11.78	<0.001
Men	9	47.3 ± 8.6	35.06 (33.16, 36.39)	34.47 ± 5.83	<0.05	114.22 ± 22.36	109.13 ± 21.98	<0.05

The distribution of variables was evaluated using the Shapiro–Wilk test. Data with a normal distribution are expressed as the mean ± standard deviation (SD), while non-normally distributed variables are reported as the median with interquartile range (IQR). A *p*-value of less than 0.05 was deemed statistically significant. Abbreviations and formulae: BMI—body mass index, calculated as weight divided by height squared (kg/m^2^); BW—body weight; SD—standard deviation; Q1—first quartile; Q3—third quartile.

**Table 2:** Baseline laboratory parameters of the study population before and after the study.

Parameter	Prior to Intervention			Subsequent to Intervention			*p*-Value	Reference Range/Desired Value
	Mean	SD		Mean		SD		
TCh (mg/dL)	189.45	44.25		174.66		50.86	0.22	<190
LDL (mg/dL)	104.27	43.94		96.91		46.95	0.55	<135
HDL (mg/dL)	55.34	15.84		52.99		13.68	0.11	>60
TG (mg/dL)	151.09	86.91		121.44		58.47	<0.05	<150
nHDL (mg/dL)	134.10	44.93		121.67		48.82	0.32	<145
UA (mg/dL)	6.36	1.46		6.08		1.47	0.09	2.40–5.70
Cr (mg/dL)	0.87	0.13		0.89		0.15	0.21	0.51–0.95
PLT (10^3^/μL)	267.89	62.25		289.39		67.83	0.11	130–400
	Median	Q1	Q3	Median	Q1	Q3		
ALT (UI/mL)	30.20	22.15	47.80	28.15	21.53	43.33	0.07	<35.0
AST (UI/mL)	23.75	20.78	33.45	24.55	20.35	33.48	0.52	<35.0
GGT (UI/mL)	27.90	20.93	39.73	22.85	16.19	35.13	<0.05	<40
Bil (mg/dL)	0.48	0.39	0.72	0.46	0.37	0.59	<0.05	0.30–1.20
ALP (UI/mL)	63.00	60.00	79.25	65.00	55.50	75.25	0.06	35–104
HbA1c (%)	5.65	5.33	5.93	5.53	5.23	5.95	<0.05	4.80–5.90
Insulin (µU/mL)	17.30	9.62	26.65	18.25	11.87	24.43	0.56	2.6–24.9
Glu (mg/dL)	98.45	88.90	105.00	90.85	83.48	100	<0.05	70.00–99.00

The distribution of variables was evaluated using the Shapiro–Wilk test. Data with a normal distribution are expressed as the mean ± standard deviation (SD), while non-normally distributed variables are reported as the median with interquartile range (IQR). Abbreviations: TCh—Total Cholesterol, LDL—Low-Density Lipoprotein Cholesterol, HDL—High-Density Lipoprotein Cholesterol, TG—Triglycerides, nHDL—non-HDL Cholesterol, Cr—Creatinine, UA—Uric Acid, PLT—Platelets, ALT—Alanine Aminotransaminase, AST—Aspartate Aminotransferase, GGT—Gamma-glutamyl Transferase, Bil—Bilirubine, ALP—Alkaline Phosphatase, HbA1c—Glycated Hemoglobin, Glu—Glucose.

**Table 3:** Impact of Glucagon-Like Peptide-1 (GLP-1) Receptor Agonists Liraglutide on Hepatic Fibrosis as Assessed by Transient Elastography (FibroScan^®^).

Parameter	Prior to Intervention			Subsequent to Intervention			*p*-Value
	Median	Q1	Q3	Median	Q1	Q3	
E (kPa)	5.60	3.90	6.45	5.20	3.60	6.00	<0.05
Fibrosis stage (F)	1.0	1.0	0.0	1.0	1.0	0.0	0.18

The distribution of variables was evaluated using the Shapiro–Wilk test. Data with non-normally distributed variables are reported as the median with interquartile range (IQR). A *p*-value of less than 0.05 was deemed statistically significant. Abbreviations: E—liver stiffness, F—fibrosis stage.

**Table 4:** Effect of Glucagon-Like Peptide-1 (GLP-1) Receptor Agonist Liraglutide on Fibrosis Parameters and Predictive Models.

Parameter	Prior to Intervention			Subsequent to Intervention			*p*-Value
	Mean	SD		Mean	SD		
ELF	6.71	0.24		6.63	0.25		<0.05
	Median	Q1	Q3	Median	Q1	Q3	
Fib-4	0.84	0.64	1.26	0.81	0.62	1.16	0.09
APRI	0.32	0.25	0.51	0.32	0.24	0.41	0.06
BARD	1.00	1.00	3.0	2.50	1.00	3.00	0.24
AST/ALT	0.75	0.71	0.97	0.87	0.73	0.93	0.06

The distribution of variables was evaluated using the Shapiro–Wilk test. Data with a normal distribution are expressed as the mean ± standard deviation (SD), while non-normally distributed variables are reported as the median with interquartile range (IQR). Abbreviations: ELF—Enhanced Liver Fibrosis, E—liver stiffness, F—fibrosis—F0/F1, Fib-4—fibrosis-4 score, APRI—AST to Platelet Ratio Index, BARD—BARD score (BMI, ALT, AST, diabetes values), AST/ALT—AST/ALT ratio.

**Table 5:** Impact of glucagon-like peptide-1 (GLP-1) Receptor agonist liraglutide on markers of fibrosis.

Parameter	Prior to Intervention			Subsequent to Intervention			*p*-Value
	Median	Q1	Q3	Median	Q1	Q3	
PIIINP (ng/mL)	243.80	168.35	341.95	192.75	139.10	301.48	0.06
TIMP1 (µg/mL)	129.35	99.50	150.90	131.35	94.25	145.15	0.16
HYA (ng/mL)	130.70	115.05	149.45	126.20	112.08	149.78	0.26
TGF-α (pg/mL)	165.30	146.20	183.45	192.90	166.40	211.95	0.68
HGF (pg/mL)	525.90	448.08	664.65	511.75	478.73	650.18	0.36
CHI3L1 (pg/mL)	1122.20	1006.4	1508.7	1231.95	1070.1	1531.5	0.66

The distribution of variables was evaluated using the Shapiro–Wilk test. Data with non-normally distributed variables are reported as the median with interquartile range (IQR). A *p*-value of less than 0.05 was deemed statistically significant. Abbreviations: PIIINP—Procollagen III N-Terminal Propeptide, TIMP1—Tissue Inhibitor of Metalloproteinases 1, CHI3L1—Chitinase-3-like Protein 1, HYA—Hyaluronic Acid, TGF-α —Transforming Growth Factor Alpha, HGF—Hepatocyte Growth Factor.

## Data Availability

Data are available from the corresponding authors for researchers with a justified request.

## Abbreviations

The following abbreviations are used in this manuscript:

AASLD	American Association for the Study of Liver Diseases
ALD	Alcohol Related Liver Disease
ALP	Alkaline Phosphatase
ALT	Alanine Aminotransferase
a2M	Alpha-2-Macroglobulin
APOA1	Apolipoprotein A1
AST	Aspartate Aminotransferase
APRI	Aspartate Aminotransferase to Platelet Ratio Index
BARD	BMI, AST/ALT ratio, and Diabetes
Bil	Bilirubin
BMI	Body Mass Index
BW	Body Weight
CAP	Controlled Attenuation Parameter^®^
CHI3L1	Chitinase-3-like Protein 1
EASD	European Association for the Study of Diabetes
EASL	European Association for the Study of the Liver
EASO	European Association for the Study of Obesity
EMA	European Medicines Agency
ELF test	Enhanced Liver Fibrosis Test
FIB-4	Fibrosis-4 Index
GGT	Gamma-glutamyl Transferase
GLP-1	Glucagon-Like Peptide-1
GLP-1 RA	Glucagon-Like Peptide-1 Receptor Agonist
HDL	High-Density Lipoprotein
HDL-c	High-Density Lipoprotein cholesterol
HGF	Hepatocyte Growth Factor
HOMA-IR	Homeostasis Model Assessment of Insulin Resistance
HYA	Hyaluronic Acid
IFN-γ	Interferon Gamma
LDL	Low-Density Lipoprotein cholesterol
MASH	Metabolic Dysfunction-Associated Steatohepatitis
MASL	Metabolic Dysfunction-Associated Steatotic Liver
MASLD	Metabolic Dysfunction-Associated Steatotic Liver Disease
Met-ALD	MASLD with Moderate Alcohol Consumption
METS-IR	Metabolic Score for Insulin Resistance
MRI-PDFF	MRI Proton Density Fat Fraction
NAFLD	Non-Alcoholic Fatty Liver Disease
nHDL	Non-HDL Cholesterol
PIIINP	N-terminal Propeptide of Type III Procollagen
QUICKI	Quantitative Insulin Sensitivity Check Index
TGF-α	Transforming Growth Factor-alpha
TIMP1	Tissue Inhibitor of Metalloproteinases 1
TCh	Total Cholesterol
TyG	Triglyceride-Glucose index
TG	Triglycerides
TNF-α	Tumor Necrosis Factor alpha
UA	Uric Acid
WHR	Waist-to-Hip Ratio
